# Crystal Engineering of Nucleic Acid Macro-molecules using Selenium Atoms

**DOI:** 10.1063/4.0000800

**Published:** 2025-10-27

**Authors:** Zhen Huang, Hehua Liu, Jianhua Gan

**Affiliations:** 1 SeNA Research Institute, College of Life Sciences, Hubei University, Wuhan, Hubei, China; 2 School of Life Sciences, Fudan University, Shanghai, Shanghai, China

## Abstract

Nucleic acids play important roles in life processes, such as genetic material, gene regulation, protein synthesis, signaling, catalysis, and viral infection. Nucleic acids are considered as ones of the keys to understand the cellular functions and disease mechanisms. Nucleic acid structure biology, especially DNA and RNA X-ray crystallography, can greatly accelerate the studies of nucleic acid-protein structures, functions and mechanisms. However, due to the crystallization, heavy-atom derivatization and phase determination, X-ray crystallography of nucleic acids (DNA and RNA, and their protein complexes) is challenging and the structures of many nucleic acids and their protein complexes haven’t been resolved. To address these challenges, we have developed the selenium-atom specifically derivatized nucleic acids (SeNA), which can not only enhance the derivatization and diffraction phase determination, but also facilitate crystal growth without significant structure perturbation. This method has huge advantages over the traditional methods, such halogen derivatizations, heavy-metal socking, and molecular replacement. Recently, our laboratory has been further focusing on selenium nucleic acid (a new paradigm of nucleic acids), exploring nucleic acid molecules at the atomic level, and expanding its potential applications in 3D structure-and-function studies, gene re-design, bio-informatics, molecular diagnostics and bio-therapeutics, including mRNA, antisense, siRNA and other potential nucleic

